# Differential in vitro effects of targeted therapeutics in primary human liver cancer: importance for combined liver cancer

**DOI:** 10.1186/s12885-022-10247-6

**Published:** 2022-11-19

**Authors:** Ihtzaz Ahmed Malik, Mansi Rajput, Rieke Werner, Dorothea Fey, Niloofar Salehzadeh, Christine A. F. von Arnim, Jörg Wilting

**Affiliations:** 1 Department of Geriatrics, University Medical Center Goettingen, Waldweg 33, D-37073 Goettingen, Germany; 2 Department of Anatomy and Cell Biology, University Medical Center Goettingen, Kreuzbergring 36, 37075 Goettingen, Germany

**Keywords:** Cholangiocellular carcinoma, Hepatocellular carcinoma, Combined liver cancer, AKT pathway, Kinase inhibitors

## Abstract

The incidence of primary liver tumors, hepatocellular carcinoma (HCC), intrahepatic cholangiocellular carcinoma (ICC), and combined HCC/ICC (cHCC/CC) is increasing. For ICC, targeted therapy exists only for a small subpopulation of patients, while for HCC, Sorafenib and Lenvatinib are in use. Diagnosis of cHCC/CC is a great challenge and its incidence is underestimated, bearing the risk of unintended non-treatment of ICC. Here, we investigated effects of targeted inhibitors on human ICC cell lines (HUH28, RBE, SSP25), in comparison to extrahepatic (E)CC lines (EGI1, CCC5, TFK1), and HCC/hepatoblastoma cell lines (HEP3B, HUH7, HEPG2). Cells were challenged with: AKT inhibitor MK-2206; multikinase inhibitors Sorafenib, Lenvatinib and Dasatinib; PI3-kinase inhibitors BKM-120, Wortmannin, LY294002, and CAL-101; and mTOR inhibitor Rapamycin. Dosage of the substances was based on the large number of published data of recent years. Proliferation was analyzed daily for four days. All cell lines were highly responsive to MK-2206. Thereby, MK-2206 reduced expression of phospho(p)-AKT in all ICC, ECC, and HCC lines, which mostly corresponded to reduction of p-mTOR, whereas p-ERK1/2 was upregulated in many cases. Lenvatinib showed inhibitory effects on the two HCC cell lines, but not on HEPG2, ICCs and ECCs. Sorafenib inhibited proliferation of all cells, except the ECC line CCC5. However, at reduced dosage, we observed increased cell numbers in some ICC experiments. Dasatinib was highly effective especially in ICC cell lines. Inhibitory effects were observed with all four PI3-kinase inhibitors. However, cell type-specific differences were also evident here. Rapamycin was most effective in the two HCC cell lines. Our studies show that the nine inhibitors differentially target ICC, ECC, and HCC/hepatoblastoma lines. Caution should be taken with Lenvatinib and Sorafenib administration in patients with cHCC/CC as the drugs may have no effects on, or might even stimulate, ICC.

**Supplementary Information:**

The online version contains supplementary material available at 10.1186/s12885-022-10247-6.

## Introduction

Hepatocellular carcinoma (HCC) and cholangiocellular carcinoma (CCC) are the most common primary hepatic malignancies, with 841,000 new cases worldwide in 2018, out of which 75%-85% accounted for HCC and 10%-15% for CCC [1, 2]. CCC is a relatively rare tumor in Western countries, but with an increasing incidence and mortality rate in recent decades. CCC is an aggressively growing tumor, which, despite general advances in diagnosis and therapy, still has a poor prognosis. CCC originates from epithelial cells of the bile ducts. According to the anatomical localization, they are further classified as intrahepatic CCC (ICC) and extrahepatic CCC (ECC). Thereby, ECC comprises the perihilar CCC (pCC) and the distal CCC (dCC) [2–4].

The prognosis for liver cancer patients is very poor. For example, in the United States of America, the 5-year survival rate for patients with HCC has remained at a low 18% [5]. The situation is even worse for ICC, where the 5-year survival rate is just 8% [6]. Approximately 20–25% of ICC cases are discovered incidentally. Surgical resection is a potential curative option but often difficult to perform due to tumor size and anatomical location [7], making chemotherapy with gemcitabine and cisplatin the next line of treatment [8]. Targeted therapy with Pemigatinib and Infigratinib was approved for patients with unresectable, locally advanced CCC with *Fibroblast growth factor receptor-2* gene mutation or fusion, which only represent 10–15% of ICC patients [9, 10]. Individual targeted therapy may be offered in some clinics based on genetic screening of the tumor. For targeted therapy of HCC, Sorafenib [11], and the recently approved Lenvatinib [12], are applied as first-line treatment options. However, these multi-kinase inhibitors improve the overall survival of patients marginally above 3 months, and their high toxicity is a serious challenge[11, 12].

A particular problem of primary liver cancer is the occurrence of combined HCC/ICC (cHCC/CC). Its incidence is reported to be highly variable. Due to great diagnostic challenge, its occurrence is likely to be massively underestimated. Of note, a single pass biopsy of a non-representative area will result in an incorrect diagnosis [13]. Besides the classical type of cHCC/CC, a less common type with stem cell features is classified by the WHO [14].

In both HCC and the small number of cHCC/CC tested, tumor protein *TP53* is among the most frequently mutated genes [15, 16]. This also holds true for ECC [17, 18]. TP53 is a central tumor suppressor and is regulated by numerous signaling pathways, including the mitogen-activated protein kinase (MAPK) and PI3K/AKT pathway [19]. Oncogenic activation of the MAPK pathway, as well as the RAS and MET pathways were also found in ICC [20]. The PI3K/AKT/mTOR and the RAS/RAF/MEK/ERK signaling pathways are involved in various cancers, and play important roles in proliferation, angiogenesis, and cell survival. The PI3K/AKT/mTOR signaling cascade has been intensively studied and high activation of PI3K signaling pathways has been observed in ICC [21], compared to healthy bile duct epithelium [22].

There are a considerable number of studies and clinical trials on targeted drugs in HCC and CCC. (We cannot cite all of these studies; therefore, for review see: [18, 23, 24]). In the vast majority of cases, these studies focus on specific tumor entities, HCC or CCC. However, a considerable number of cHCC/CC occur, which may not be detected by clinical diagnosis. Part of the tumor may therefore not respond to the selected targeted drug and might even have a growth advantage. Therefore, we performed a comprehensive in vitro study on the effects of targeted drugs on HCC/hepatoblastoma, ICC and ECC cell lines. We applied nine molecular inhibitors (including the approved drugs Sorafenib and Lenvatinib), to investigate tumor-specific and tumor type-overlapping effects. The latter will possibly be applicable to cHCC/CC.

## Material and methods

### Materials

If not mentioned otherwise, all chemicals and reagents were purchased from Sigma-Aldrich (St. Louis, USA) and Merck (Darmstadt, Germany). All inhibitors were purchased from Selleckchem (Munich, Germany). Inhibitors were: Sorafenib, Lenvatinib (multi-kinase inhibitor), Dasatinib (tyrosine-kinase inhibitor), BKM120, Wortmannin, LY294002, and CAL-101 (PI3K inhibitors), MK-2206 (AKT inhibitor), and Rapamycin (mTOR inhibitor). The inhibitors were dissolved in dimethyl sulfoxide (DMSO) (Sigma Aldrich, Taufkirchen, Germany). 

### Cell culture

The cells were grown in appropriate medium (listed in Table 1) in a humidified incubator at 37 °C and 5% CO_2_.

**Table 1:** Cell lines used in the study

Cell lines	Year of deposit	Medium	Tumor	Distributor
HUH28	2004	RPMI 1640, 10% FCS, Penicillin–Streptomycin	ICC	Cell Bank, Riken BioResources Center, Tsukuba, Japan
RBE	1996	RPMI 1640, 10% FCS, Penicillin–Streptomycin	ICC	Cell Bank, Riken BioResources Center, Tsukuba, Japan
SSP25	1996	RPMI 1640, 10% FCS, Penicillin–Streptomycin	ICC	Cell Bank, Riken BioResources Center, Tsukuba, Japan
EGI1	1984	RPMI 1640, 10% FCS, Penicillin–Streptomycin	ECC	DSMZ, Leibniz Institute, Braunschweig, Germany
CCC5	2015	DMEM, Keratinocyte, 20% FCS, Penicillin–Streptomycin	ECC	DSMZ, Leibniz Institute, Braunschweig, Germany
TFK1	2007	RPMI 1640, 10% FCS, Penicillin–Streptomycin	ECC	DSMZ, Leibniz Institute, Braunschweig, Germany
HEP3B	1976	RPMI 1640, 10% FCS, Penicillin–Streptomycin	HCC	DSMZ, Leibniz Institute, Braunschweig, Germany
HUH7	1982	RPMI 1640, 10% FCS, Penicillin–Streptomycin	HCC	Originally from Cell Bank, Riken BioResources Center, Tsukuba, Japan (via Cell lines service GmbH, Eppelheim, Germany)
HEPG2	1975	RPMI 1640, 10% FCS, Penicillin–Streptomycin	Hepato-blastoma	DSMZ, Leibniz Institute, Braunschweig, Germany

A series of experiments were conducted on common ICC (HUH28, RBE, SSP25), ECC (EGI1, CCC5, TFK1), HCC (HEP3B, HUH7) and hepatoblastoma (HEPG2) cell lines over a course of 96 h using crystal violet proliferation assays. Nine potential inhibitors including Sorafenib and Lenvatinib, which are FDA approved drugs to treat HCC, were used. Based on literature and our previous studies [25], each drug dissolved in DMSO, was used in two concentrations. Adherent cells were analyzed at a regular interval of 24 h for 4 days. Cells incubated in the solvent DMSO served as controls in accordance to the highest concentration used in the test group.

### Proliferation assay

Proliferation assays were performed as described [25]. Shortly, 5000 cells per well were seeded into 96-well-cell culture plates, and allowed to adhere overnight in the incubator at 37 °C. The cells were treated with inhibitors in each two different concentrations over a period of 96 h. Adherent cells were fixed with 5% glutaraldehyde after 0, 24, 48, 72, and 96 h and stained with crystal violet (0.1% in deionized water). 10% acetic acid was added before absorption was measured at 570 nm with iMark™ microplate reader (Bio-Rad). Data are presented as the mean value of n = 3-5 independent experiments with each 6-8 replicates.

### Intracellular signaling after AKT inhibition

500,000 cells per well were seeded into 6-well-culture plates, and allowed to adhere overnight at 37 °C. The cells were treated with 25 µM MK-2206 diluted in DMSO for 3, 8, and 24 h. Controls were performed with DMSO, in accordance to the highest concentration used in the test group. The wells were rinsed with washing buffer followed by lysis buffer. The wells were scraped with a cell scraper and the cell lysate was collected for Western blots.

### Protein extraction and Western blot analysis

Protein extraction and Western blot were performed as described previously [26]. Cells were lysed using lysis buffer (1 mM sodium orthovanadate and protease inhibitor cocktail in RIPA buffer) to form a lysate. Protein extraction was performed by centrifuging the lysate at 4 °C at 20,800 rcf (Eppendorf Centrifuge 5417R) for 15 min. Bradford protein assay was performed to measure the total protein concentration. The absorption was measured at 595 nm with iMark™ microplate reader (Bio-Rad). For Western blot analysis, 50 µg of protein was loaded on polyacrylamide gels (SurePAGE™ 4–12% Bis–Tris Gel, Genscript Biotech, New Jersey, USA) under reducing conditions. After electrophoresis, proteins were transferred to PVDF-Roti Membrane (Carl Roth, Karlsruhe, Germany). The membrane was incubated with primary antibodies overnight at 4 °C followed by horseradish-peroxidase conjugated secondary antibody with intermediate washing of membrane with washing buffer (TBS with 0.1% tween-20). Immunodetection was carried out with ChemiDoc™ MP Imaging System (Bio-Rad) using antibodies and concentrations listed in Table 2.

**Table 2:** Antibodies used for Western blotting

Antibody	Company	Catalog number	WB
AKT rabbit mAb	Cell signaling	4691	1:1000
p-AKT (Ser473) rabbit pAb	Cell signaling	9271	1:1000
mTOR rabbit mAb	Cell signaling	2983	1:1000
p-mTOR (Ser2448) rabbit mAb	Cell signaling	5536	1:1000
ERK1/2 rabbit pAb	Cell signaling	9102	1:1000
p-ERK1/2 (Thr202/Tyr204) rabbit mAb	Cell signaling	4370	1:2000
α- tubulin mouse mAb (HRP)	Abcam	ab40742	1:10,000
gt-anti-rb IgG peroxidase	Sigma	A6154	1:2000

### Statistical analyses

The data were analyzed using Microsoft® Excel 2019 and Graph pad Prism version 4 software (San Diego, USA). All experimental errors were calculated as SEM. Statistical significance was calculated with one-way analysis of variance (ANOVA) between treatment and control groups at all time points. Statistical level of significance was accepted at *p ≤ 0.05, **p ≤ 0.01, and ***p ≤ *0.001* with 95% level of confidence.

## Results

### Reduction in cell proliferation in ICC cell lines by AKT inhibitor MK-2206

**Fig. 1:**
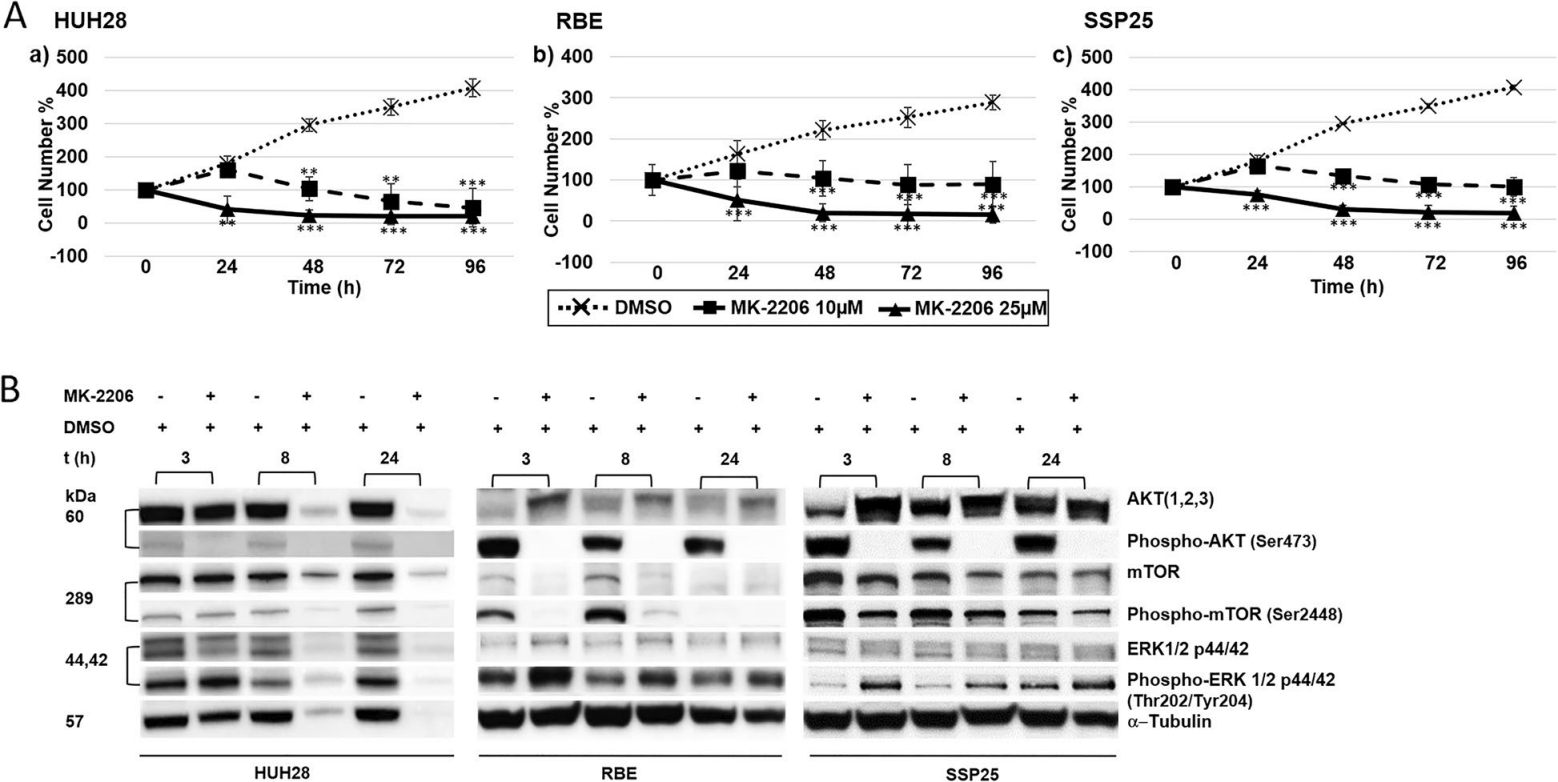
Proliferation studies and Western-blot analysis of ICC cell lines. **A** Effects of AKT inhibitor MK-2206 on HUH28 (a), RBE (b), and SSP25 (c) after 0, 24, 48, 72 and 96 h (h). 0 h values were set to 100%. Concentrations of MK-2206 are indicated. Shown are the mean values of n = 3–5 independent experiments with each 6–8 replicates. Controls were treated with solvent (DMSO). Significance was tested by one-way ANOVA between the control and treatment groups. *p < 0.05, **p < 0.01, ***p < 0.001 with 95% level of confidence. **B** Cropped Western blot images of 25 µM MK-2206-treated HUH28 and RBE and SSP25 with specific antibodies against total AKT and p-AKT, mTOR and p-mTOR, ERK1/2 and p-ERK1/2 after 3, 8 and 24 h. The samples were derived from the same experiment and the blots were processed parallelly. α-Tubulin was used as loading control*.* Note reduced phosphorylation of AKT and mTOR and increase in p-ERK1/2, in addition to death of HUH28 cells after 8 h and 24 h of MK-2206

Our results show that the ICC cell lines (HUH28, RBE, SSP25) incubated only in cell culture media and media containing DMSO increased in number in an almost linear fashion. Highly significant inhibition was observed with MK-2206, where we noticed a significant reduction in cell proliferation in a dose dependent manner. The cell numbers were reduced up to 95% compared to initial cell number (Fig. 1A).

By Western blot analysis, distinct down-regulation of p-AKT (Ser473) was noticed at 3—24 h in all of the studied ICC cell lines after MK-2206 treatment (Fig. 1B & Suppl. Fig. 1). Similar but less consistent observations were made with p-mTOR (Ser2448), a downstream target of AKT. In contrast, an increased phosphorylation of ERK1/2 was visible after 3 h in all investigated cells lines, the signal intensity remained above the control until 24 h in RBE and SSP25, while HUH28 showed signs of cell death after 8 h of MK-2206 treatment.

### Reduction in cell proliferation in ECC cell lines by AKT inhibitor MK-2206

**Fig. 2:**
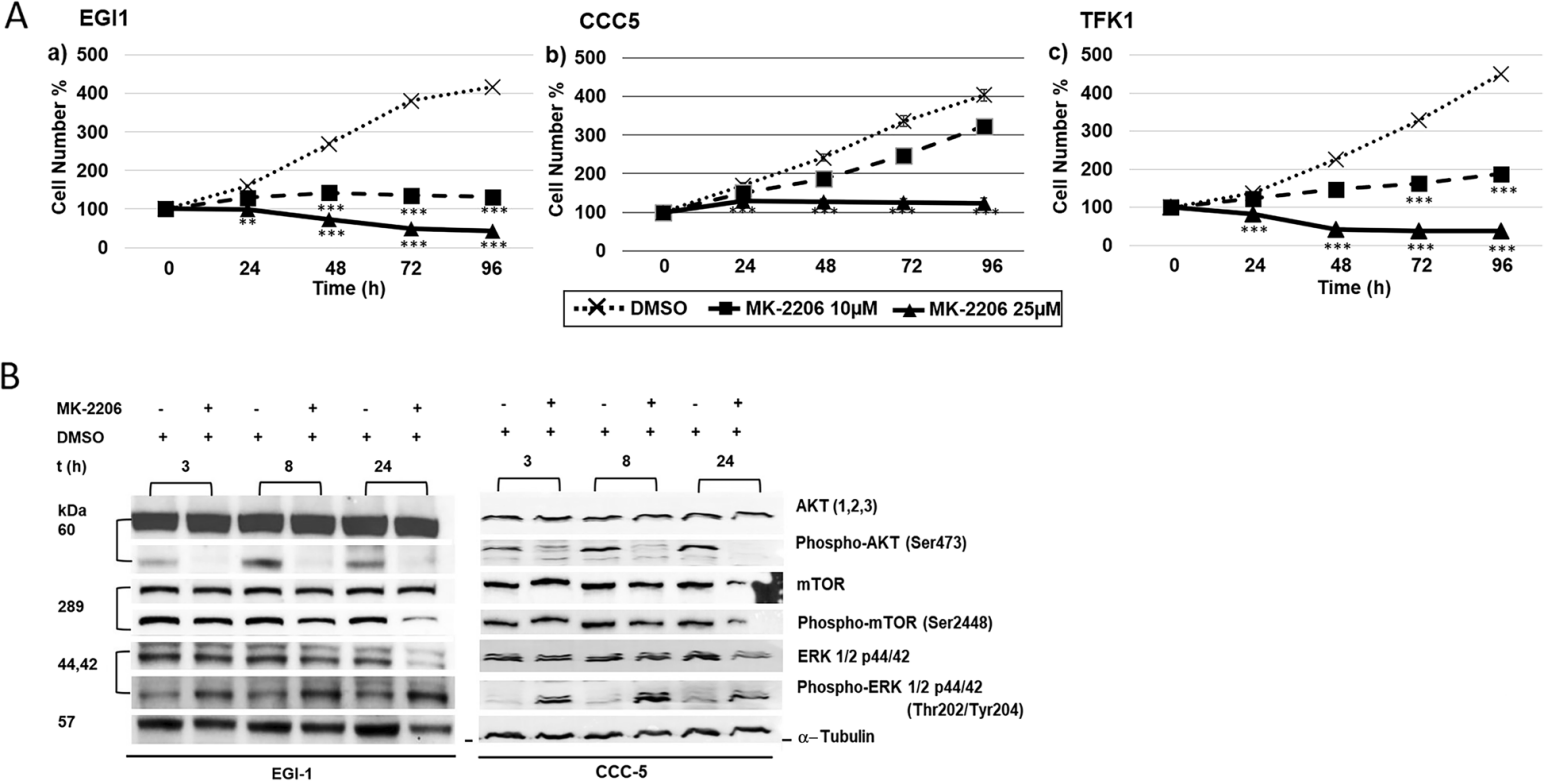
Proliferation studies and Western-blot analysis of ECC cell lines. **A** Effects of AKT inhibitor MK-2206 on EGI1 (a), CCC5 (b), and TFK1 (c) after 0, 24, 48, 72 and 96 h (h). 0 h values were set to 100%. Concentrations of MK-2206 are indicated. Shown are the mean values of n = 3–5 independent experiments with each 6–8 replicates. Controls were treated with solvent (DMSO). Significance was tested by one-way ANOVA between the control and treatment groups. *p < 0.05, **p < 0.01, ***p < 0.001 with 95% level of confidence. **B** Cropped Western blot images of 25 µM MK-2206-treated EGI1 and CCC5 with specific antibodies against total AKT and p-AKT, mTOR and p-mTOR, ERK1/2 and p-ERK1/2 after 3, 8 and 24 h. α-Tubulin was used as loading control*.* Note reduced phosphorylation of AKT, and increase in p-ERK1/2

In parallel to ICC cell line, our studies also showed a highly significant effect of 10 µM MK-2206 on ECC cell lines EGI1 and TFK1. However, CCC5 was less susceptible (Fig. 2A).

By Western blot analysis, down-regulation of p-AKT was clearly visible at 3, 8 and 24 h in EGI1 and CCC5 after MK-2206 treatment. Results for p-mTOR were less distinct, while up-regulation of p-ERK1/2 was visible after 3–24 h in the two cell lines tested (Fig. 2B & Suppl. Fig. 2).

### Reduction in cell proliferation in HCC/hepatoblastoma cell lines by AKT-inhibitor MK-2206

**Fig. 3:**
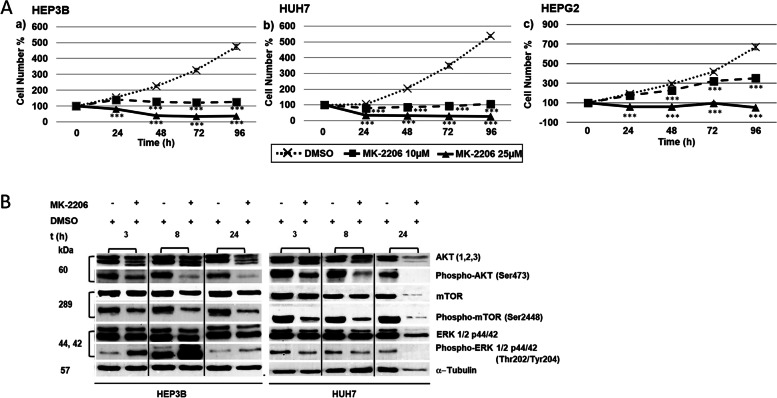
Proliferation studies and Western-blot analysis of HCC/hepatoblastoma cell lines. **A** Effects of AKT inhibitor MK-2206 on HEP3B (a), HUH7 (b), and HEPG2 (c) after 0, 24, 48, 72 and 96 h (h). 0 h values were set to 100%. Concentrations of MK-2206 are indicated. Shown are the mean values of n = 3–5 independent experiments with each 6–8 replicates. Controls were treated with solvent (DMSO). Significance was tested by one-way ANOVA between the control and treatment groups. *p < 0.05, **p < 0.01, ***p < 0.001 with 95% level of confidence. **B** Cropped Western blot images of 25 µM MK-2206-treated HEP3B and HUH7 with specific antibodies against total AKT and p-AKT, mTOR and p-mTOR, ERK1/2 and p-ERK1/2 after 3, 8 and 24 h. The samples were derived from the same experiment and the blots were processed parallelly. α-Tubulin was used as loading control*.* Note reduced phosphorylation of AKT and mTOR, and heterogenous results with p-ERK1/2

In controls, cell numbers increased in an almost linear fashion during 4 days of incubation. Both 10 µM and 25 µM MK-2206 showed significant inhibitory effects in all three cell lines (HEP3B, HUH7 and HEPG2). We noticed a reduction of about 90% at 96 h treatment with 25 µM MK-2206 in comparison to respective controls (Fig. 3A).

By Western blot analysis we observed reduced expression of p-AKT and p-mTOR after treatment with 25 µM MK-2206 in HEP3B and HUH7, while results for p-ERK1/2 were heterogenous (Fig. 3B & Suppl. Fig. 3).

### Changes in cell proliferation using multikinase, PI3K and mTOR inhibitors

Dose- and cell line-dependent effects were noted with multikinase inhibitors Sorafenib and Lenvatinib, and the tyrosine-kinase inhibitor Dasatinib.

**Fig. 4:**
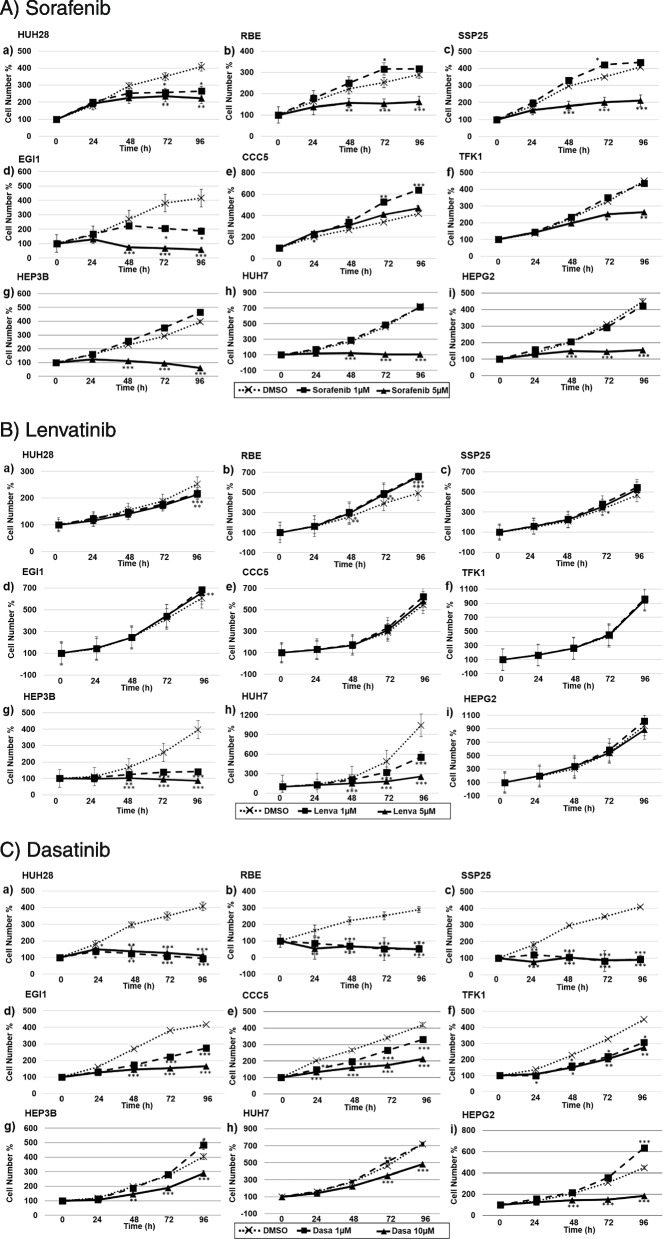
Proliferation studies of ICC (HUH28, RBE, SSP25, a-c), ECC (EGI1, CCC5, TFK1, d-f) and HCC/hepatoblastoma (HEP3B, HUH7, HEPG2, g-i) cell lines. Effects of (**A**) Sorafenib, (**B**) Lenvatinib, and (**C**) Dasatinib after 0, 24, 48, 72 and 96 h (h). 0 h values were set to 100%. Concentrations of Sorafenib are indicated. Shown are the mean values of n = 3–5 independent experiments with each 6–8 replicates. Controls were treated with solvent (DMSO). Significance was tested by one-way ANOVA between the control and treatment groups. *p < 0.05, **p < 0.01, ***p < 0.001 with 95% level of confidence

Sorafenib was effective at concentrations of 5 µM in all investigated cell lines except for the ECC cell line CCC5 and showed the maximum reduction in cell numbers with HCC and hepatoblastoma cell line HEPG2. At 1 µM, Sorafenib increased proliferation in some experiments performed with RBE and SSP25 (ICC cell lines) and CCC5 (ECC cell line) (Fig. 4A).

At 1 µM and 5 µM, Lenvatinib significantly inhibited growth of HCC cell lines HEP3B and HUH7, but not hepatoblastoma cell line HEPG2. We did not observe any inhibitory effects on ICC and ECC cells (Fig. 4B).

Dasatinib showed maximum reduction in cell numbers on ICC cells where the dosage of 1 µM was already very effective. The results for ECC and HCC cell lines were less impressive, and only the higher dose of 10 µM induced consistent reduction of cell proliferation (Fig. 4C).

**Fig. 5:**
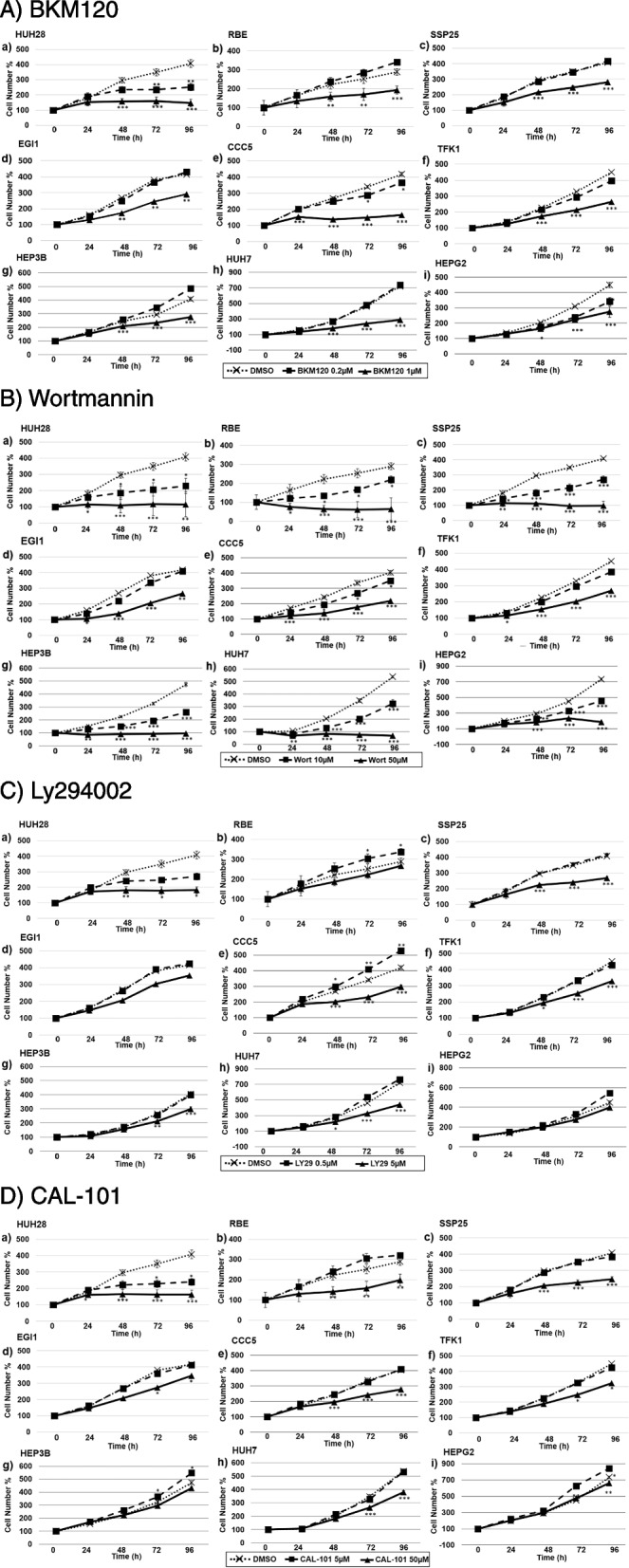
Proliferation studies of ICC (HUH28, RBE, SSP25, a-c), ECC (EGI1, CCC5, TFK1, d-f) and HCC/hepatoblastoma (HEP3B, HUH7, HEPG2, g-i) cell lines. Effects of (**A**) BKM120, (**B**) Wortmannin (Wort), (**C**) LY294002 (LY29), and (**D**) CAL-101 after 0, 24, 48, 72 and 96 h (h). 0 h values were set to 100%. Concentrations of BKM120 are indicated. Shown are the mean values of n = 3–5 independent experiments with each 6–8 replicates. Controls were treated with solvent (DMSO). Significance was tested by one-way ANOVA between the control and treatment groups. *p < 0.05, **p < 0.01, ***p < 0.001 with 95% level of confidence

Since tumor suppressors of the PIK3 pathway such as PTEN (phosphatase and tensin homolog) were found to be mutated in combined HCC/ICC (Cosmic, 2021), we tested four different PI3K inhibitors, three of them inhibit predominantly PIK3CA (BKM120, Wortmannin, and LY294002) and one PIK3CD (CAL-101). The effects were relatively similar. PIK3CA inhibitors BKM-120 and Wortmannin showed significant inhibitory effects at higher concentrations on all cell lines, while LY294002 at higher dose had significant effect on all cell lines but RBE (ICC cell line), EGI1 (ECC cell line), and HEPG2 (hepatoblastoma cell line). The effects of CAL-101 were moderate (Figs. 5A-D). The most prominent results were obtained with BKM120 and Wortmannin in all cell lines tested.

**Fig. 6:**
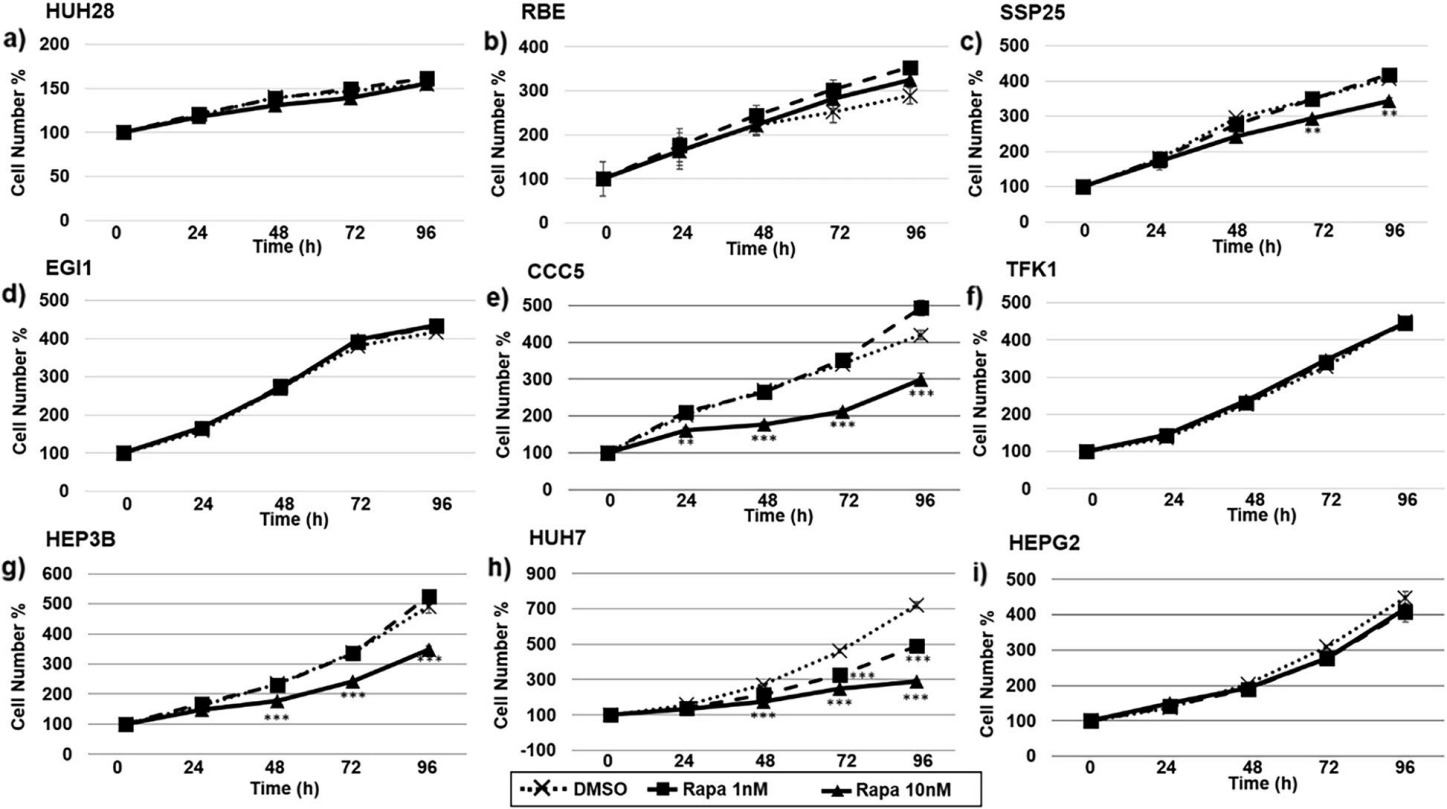
Proliferation studies of ICC (HUH28, RBE, SSP25, **a**-**c**, ECC (EGI1, CCC5, TFK1, **d**-**f** and HCC/hepatoblastoma (HEP3B, HUH7, HEPG2, **g**-**i** cell lines. Effects of Rapamycin (Rapa) after 0, 24, 48, 72 and 96 h (**h**). 0 h values were set to 100%. Concentrations of Rapamycin are indicated. Shown are the mean values of n = 3–5 independent experiments with each 6–8 replicates. Controls were treated with solvent (DMSO). Significance was tested by one-way ANOVA between the control and treatment groups. *p < 0.05, **p < 0.01, ***p < 0.001 with 95% level of confidence

Finally, we studied the mTOR inhibitor Rapamycin. At a concentration of 10 nM, Rapamycin could significantly reduce the cell numbers of CCC5 (ECC cell line), HEP3B and HUH7 (HCC cell lines), while the proliferation was unaffected in other experiments (Fig. 6).

## Discussion

We previously developed a preclinical ICC model in mice by oral application of thioacetamide (TAA) in drinking water. Besides typical cirrhosis we observed development of ICC after 22 weeks of TAA [27]. Interestingly, the same mode of TAA application in rats induces both HCC and ICC [28, 29]**.** These preclinical rodent models can be used to investigate tumor specific and tumor type overlapping effects of therapeutics. Our preliminary data indicate overexpression of p-AKT in the murine TAA model (data not shown). Here, we searched for targeted drugs that inhibited *in-vitro* cell growth either in a cell type-specific or comprehensive manner.

We observed differential effects of nine inhibitors targeting the PI3K/AKT/mTOR pathway in ICC, ECC, and HCC cell lines. The PI3K/AKT pathway plays an important role in proliferation, angiogenesis, apoptosis, cytoskeletal organization, and cell survival. This pathway is also known to interact with other signaling pathways such as the RAS-RAF pathway. Consequently, alterations in the PI3K/AKT pathway affect other signaling pathways, leading to uncontrolled cell proliferation and survival and eventually tumorigenesis by inducing transcription of the most commonly mutated genes such as AT-Rich Interaction Domain 1A (ARID1), BRCA1 Associated Protein 1 (BAP1), and Polybromo 1 (PBRM1), which are downstream of the PI3K/AKT pathway [30].

The four PI3-kinase inhibitors tested (BKM-120, Wortmannin, LY294002, and CAL-101) showed promising results by significantly reducing proliferation, with some variation depending on the tumor cell type. CAL-101 showed good effects in ICC cell lines, BKM120 and Wortmannin inhibited proliferation in all cell lines effectively. Thereby, Wortmannin showed better results in ICC and HCC than in ECC. The effects of LY294002 were somewhat intermediate. Therefore, PI3-kinase appears to be a promising target for the treatment of primary liver cancers.

The AKT inhibitor MK-2206 potently blocked proliferation of all cell lines tested. This is in line with the marked down-regulation of p-AKT in the cells, whereas the expression of p-mTOR was only weakly or not at all changed in the cells. In fact, the mTOR inhibitor Rapamycin did not reveal any anti-proliferative effects in 7 out of 9 cell lines tested. In other cell types, it has been shown that AKT phosphorylates mTORC1 at Ser2448 leading to cell survival, proliferation, and metabolic changes [31]. Our observation could be due to the fact that we used a lower dose of Rapamycin in our experiments. It is possible that increasing the dose of rapamycin can trigger antiproliferative activity in these cell lines. Moreover, mTORC1 pathway has been shown to have a cross-talk with the RAS-RAF pathway. Mutations in KRAS have been found in 4% of 126 samples of cHCC/CC investigated [16]. RAS phosphorylates PI3K and the AKT inhibitor RAF, which appears to be a negative feedback loop. Then, MEK phosphorylates ERK leading to either cell cycle progression or apoptosis because ERK has a dual function [32]. We regularly observed up-regulation of p-ERK1/2 in MK-2206-treated cells, indicating a pro-apoptotic function of ERK in the cell lines tested. It is becoming more and more evident that inflammatory signaling pathways such as MAPK/ERK [33, 34] can support apoptosis in cancer [35].

While MK-2206 has a comprehensive effect in ICC, ECC, and HCC, Dasatinib was most effective in ICC and inhibited cell growth effectively already at 1 µM. Dasatinib inhibits tyrosine-kinases including cKIT and EPH, and is approved for the treatment of chronic myeloid leukemia and acute lymphatic leukemia [36, 37]. Upregulation of cKit was observed in a rat model of primary liver cancer [29]. Application of MK-2206 in cHCC/CC and Dasatinib in ICC therapy might be worth testing.

Sorafenib, which is approved for HCC, was very effective in HCC/hepatoblastoma cell lines at 5 µM, but much less effective in two of three ECC cell lines. In ICC, proliferation was decreased greatly at 5 µM, but was even increased at some time points with 1 µM. Sorafenib has a very broad spectrum of activity [11, 38], and it appears that while inhibition of some kinases may have anti-cancerogenic effects, others may have pro-cancerogenic effects, and quantity of available drug in the tissue may be a very critical aspect for successful treatment. This might explain the variability in response of patients diagnosed with HCC, but might suffer from not properly diagnosed cHCC/CC, and underlines the importance of exact histopathological diagnosis.

Lenvatinib showed inhibitory effects on HCC proliferation with both concentrations, but not in the hepatoblastoma cell line HEPG2. In ICC and ECC, however, neither the lower nor the higher concentration demonstrated any anti-tumor activity. Therefore, caution should be taken with Lenvatinib administration in cHCC/CC.

## Conclusions

In sum, our studies show that primary liver tumor cell lines show distinct but overlapping patterns of reactivity to targeted molecular therapeutics. Thereby, as in case of Sorafenib, dosage is an important matter, as insufficient quantities might even induce pro-proliferative effects. For targeted therapy, it will be important to properly diagnose the incidence of combined HCC-ICC, as ICC may not or even inversely respond to Lenvatinib and Sorafenib. Customized drug therapy may be the next step to optimize therapy, tailored according to the nature of the tumor and the individual reactivity of patient-derived tumor cells.

## Supplementary Information

**Additional file 1: Suppl. Figure 1.** Uncropped Western-blot images of 25µM MK-2206-treated HUH28 and RBE and SSP25 with specific antibodies against total AKT and p-AKT, mTOR and p-mTOR, ERK1/2 and p-ERK1/2. α-Tubulin was used as loading control. Note: Images correspond to figure 1B. **Suppl. Figure 2.** Uncropped Western-blot images of 25µM MK-2206-treated EGI1 and CCC5 with specific antibodies against total AKT and p-AKT, mTOR and p-mTOR, ERK1/2 and p-ERK1/2. α-Tubulin was used as loading control. Note: Images correspond to figure 2B. **Suppl. Figure 3.** Uncropped Western-blot images of 25µM MK-2206-treated HEP3B and HUH7 with specific antibodies against total AKT and p-AKT, mTOR and p-mTOR, ERK1/2 and p-ERK1/2. α-Tubulin was used as loading control. Note: Images correspond to figure 3B.

## Data Availability

All data are included in the manuscript.
