# Supplementary material for: Differential in vitro effects of targeted therapeutics in primary human liver cancer: importance for combined liver cancer
Source: BMC Cancer. 2022 Nov 19;22:1193. doi: 10.1186/s12885-022-10247-6 (PMC9675209; doi:10.1186/s12885-022-10247-6)
Supplement: Supplementary file 1 — Additional file 1: Suppl. Figure 1. Uncropped Western-blot images of 25µM MK-2206-treated HUH28 and RBE and SSP25 with specific antibodies against total AKT and p-AKT, mTOR and p-mTOR, ERK1/2 and p-ERK1/2. α-Tubulin was used as loading control. Note: Images correspond to figure 1B. Suppl. Figure 2. Uncropped Western-blot images of 25µM MK-2206-treated EGI1 and CCC5 with specific antibodies against total AKT and p-AKT, mTOR and p-mTOR, ERK1/2 and p-ERK1/2. α-Tubulin was used as loading control. Note: Images correspond to figure 2B. Suppl. Figure 3. Uncropped Western-blot images of 25µM MK-2206-treated HEP3B and HUH7 with specific antibodies against total AKT and p-AKT, mTOR and p-mTOR, ERK1/2 and p-ERK1/2. α-Tubulin was used as loading control. Note: Images correspond to figure 3B. [file 12885_2022_10247_MOESM1_ESM.docx]

[**Supplementary**](https://submission.nature.com/submission/ba4f1b30-fed9-4fd5-a9cf-7c1916e81ff4/file/74444749-f826-4299-9286-df6b291375c4) **Figure Legends**

**
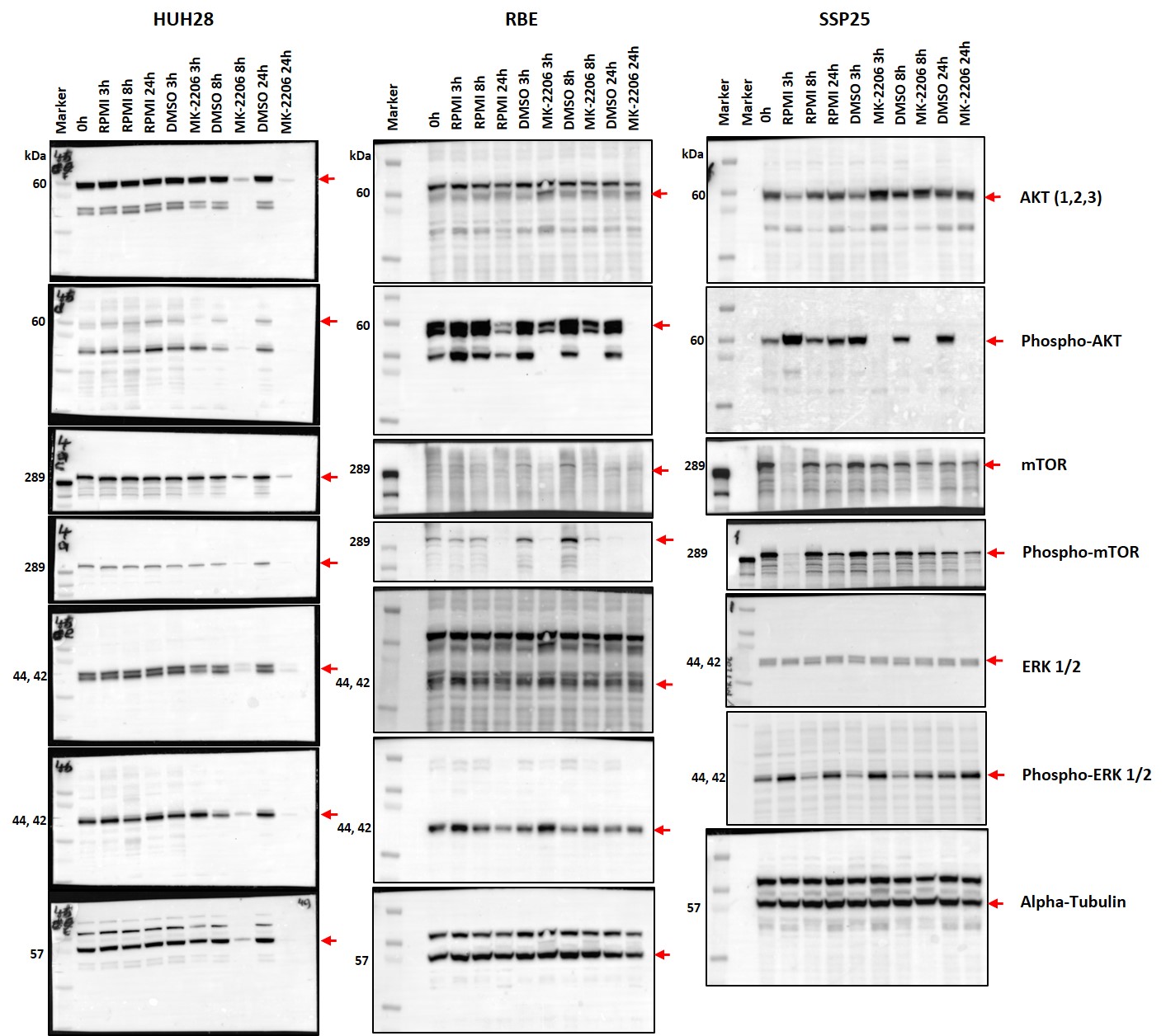
**

***Suppl. Figure 1:*** *Uncropped Western-blot images of 25µM MK-2206-treated HUH28 and RBE and SSP25 with specific antibodies against total AKT and p-AKT, mTOR and p-mTOR, ERK1/2 and p-ERK1/2. α-Tubulin was used as loading control*. Note: *Images correspond to figure 1B.*

*
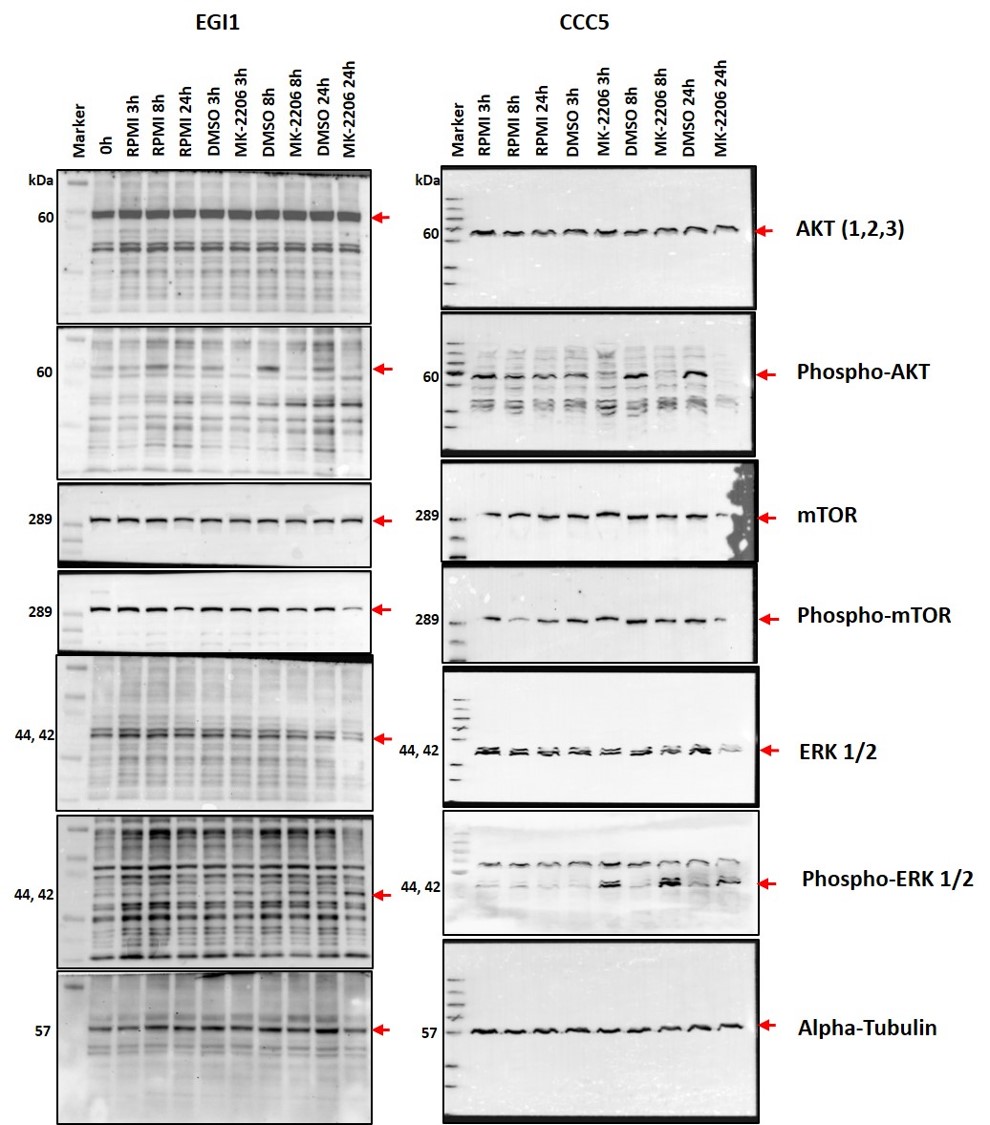
*

***Suppl. Figure 2:*** *Uncropped Western-blot images of 25µM MK-2206-treated EGI1 and CCC5 with specific antibodies against total AKT and p-AKT, mTOR and p-mTOR, ERK1/2 and p-ERK1/2. α-Tubulin was used as loading control*. Note: Images *correspond to figure 2B.*

*
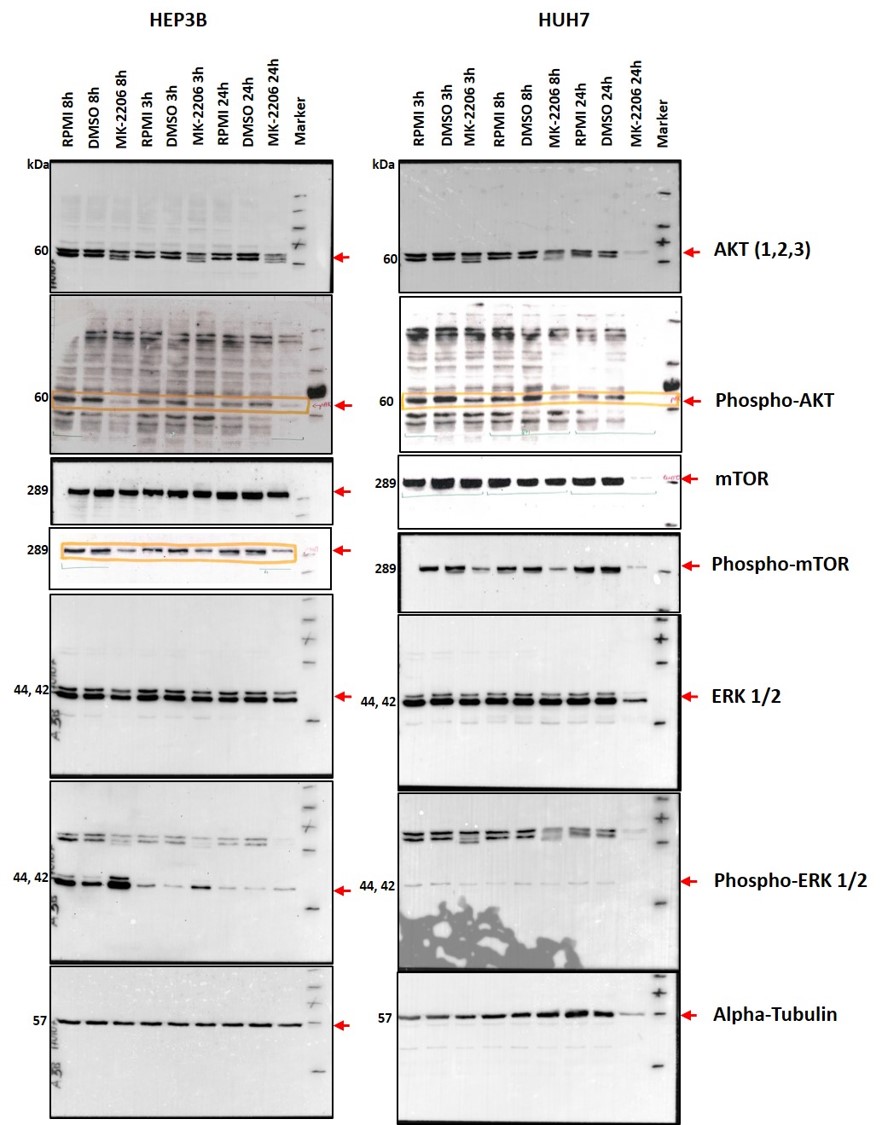
*

***Suppl. Figure 3:*** *Uncropped Western-blot images of 25µM MK-2206-treated HEP3B and HUH7 with specific antibodies against total AKT and p-AKT, mTOR and p-mTOR, ERK1/2 and p-ERK1/2. α-Tubulin was used as loading control*. Note: *Images correspond to figure 3B.*
